# Stem cell transplantation patients receiving a novel oral care protocol for oral mucositis prevention and treatment: patient-reported outcomes and quality of life

**DOI:** 10.1007/s00520-022-07073-8

**Published:** 2022-04-27

**Authors:** Monica Guberti, Stefano Botti, Andrea Fusco, Cristiana Caffarri, Silvio Cavuto, Luisa Savoldi, Nicola Serra, Francesco Merli, Michela Piredda, Maria Grazia De Marinis

**Affiliations:** 1 Department of Biomedicine and Prevention, University of Rome “Tor Vergata”, Via Montpellier 1, 00133 Rome, Italy; 2 Research and EBP Unit, Health Professions Department, Azienda USL-IRCCS Di Reggio Emilia, Via Amendola 2, 42122 Reggio Emilia, Italy; 3 Hematology Unit, Azienda USL-IRCCS Di Reggio Emilia, Via Amendola 2, 42122 Reggio Emilia, Italy; 4 Clinical Trials and Statistics Unit, SC Infrastructure, Research and Statistics, Azienda USL-IRCCS Di Reggio Emilia, Via Amendola 2, 42123 Reggio Emilia, Italy; 5 Department of Public Health, University Federico II of Naples, via S. Pansini 5, 80131 Naples, Italy; 6 Research Unit Nursing Science, University Campus Bio-Medico of Rome, Via Alvaro del Portillo 21, 00128 Rome, Italy; 7 Azienda USL-IRCCS Di Reggio Emilia, Via Amendola 2, 42122 Reggio Emilia, Italy

**Keywords:** Stem cell transplantation, Patient-reported outcomes, Oral mucositis, Quality of life, Oral mucositis daily questionnaire, Mouth and throat soreness, Pain

## Abstract

**Background:**

Oral mucositis (OM) is one of the most debilitating effects of toxicity due to hematopoietic stem cell transplantation (HSCT) conditioning regimens. The aim of this secondary analysis of the data of a phase II study designed to assess the efficacy of a novel oral care protocol containing bovine colostrum and aloe vera to prevent oral mucositis was to compare outcomes reported by patients with those collected by healthcare professionals (HCPs).

**Method:**

Data on oral mucositis severity, duration, time of onset and related pain were collected from patients using the Oral Mucositis Daily Questionnaire (OMDQ). HCPs assessed the same outcomes using the World Health Organization oral mucositis scale and pain numerical rating scale. Quality of life was assessed with the 3-level EuroQol-5 dimensions.

**Results:**

Fifty-nine autologous/allogeneic graft patients were recruited, 46 of whom (78.0%) experienced OM. Mean onset was 9.1 (SD ± 3.5) days after conditioning initiation, mean duration was 10.4 (SD ± 4.3) days, and the average maximum pain score was 3.7 (SD ± 2.7). Self-administration of the OMDQ detected oral symptoms at least 1 day sooner compared to objective assessments (*p* = 0.025). Significant differences were observed between the patient-reported and the HCP-assessment data on oral mucositis severity grading distribution (*p* < 0.0001) and highest pain score (*p* < 0.0001). Quality of life score variations were correlated with changes in oral mucositis severity during patients’ hospital stay.

**Conclusions:**

Further studies are necessary to improve the understanding of these findings; a randomised controlled trial is being set up at our institution.

## Background

Haematopoietic stem cell transplantation (HSCT) is a standard treatment for malignant and non-malignant conditions that lead to immune system failure, such as haematologic malignancies, solid tumours and autoimmune diseases [1]. Chemotherapy and/or radiotherapy-based conditioning regimens are administered prior to stem cell infusion to eradicate the underlying disease, to create space for engraftment and to provide immunosuppression [2]. Patients undergoing autologous HSCT receive their own stem cells, while those undergoing allogeneic HSCT receive stem cells from related or unrelated donors. Stem cells may be harvested from bone marrow, peripheral blood or cord blood [3].

Oral mucositis (OM), a common toxic effect of the regimen drugs used, occurs in 70–99% of HSCT patients [4, 5], and the incidence of its severe forms range from 20 to over 75%, depending on the intensity of the conditioning regimen and on the patient’s predisposing factors [6, 7]. OM results from the inflammation of the oral mucosal barrier and is accompanied by various signs and symptoms, such as erythema, ulcers, difficulty eating and/or drinking and pain [8]. Severe cases of OM are associated with extreme discomfort and may affect patients’ quality of life (QoL) and transplant-related outcomes, including infection risk and procedure-related costs [9–11].

There is limited evidence in the literature on OM prevention, and treatment is frequently symptomatic and largely based on anecdotal evidence [12, 13]. Recent evidence is available regarding the topical application of natural products, including honey, aloe vera (AV), bovine colostrum (BC) and others [14, 15]. These composite agents contain a wide variety of biologically active substances such as lactoferrin, lactoperoxidase, growth factors, immunoglobulins, cytokines, iron, folic acid, electrolytes and vitamins, which may interfere with the pathobiological pathway underlying OM [16–18]. The beneficial effects of these agents on mucosal healing have already been described as mainly attributable to their immune-modulatory, anti-inflammatory and antibacterial activity [14, 15, 19]. In addition, their topical formulas provide emollient, moisturising and hydrating effects.

The impact of OM on clinical outcomes is generally underestimated, although patients often cite OM as one of the worst side effects of their treatment [20]. For this reason, collecting patient-reported outcome measures (PROMs) as part of the patient’s clinical assessment is recommended in daily practice [21]. Increased consideration of patients’ perspectives during anticancer treatments has allowed healthcare professionals (HCPs) to improve the quality of supportive care in oncology, and PROMs are commonly assessed in clinical trials [22]. It has been shown that patient-reported assessment tools make it possible to detect symptom onset, peak and resolution earlier than HCPs’ objective evaluation [23, 24].

Knowledge of the impact of OM on patient QoL during HSCT is still limited due to the tendency to consider QoL as a secondary outcome in clinical trials which are designed to explore the toxicity of cancer treatments. Furthermore, QoL is affected by various factors during HSCT, and the assessment of its relationship with OM can be very complex [25]. The increase in the degree of OM has been associated with worse patient QoL; the adoption of strategies to prevent OM, such as low-level laser therapy (LLLT) and professional oral care, has led to improvements in QoL [11, 26].

A recent phase II study conducted by our group demonstrated that a BC–AV-based oral care protocol effectively and safely reduced the incidence of severe OM (sOM) in patients undergoing HSCT [27]. In that study, both PROMs and healthcare professional-reported outcomes (HCP-ROs) were collected to give a true picture of toxicity and its effect on QoL.

This paper reports a secondary analysis on patient-reported data with the aim to describe the variations of QoL perceived by HSCT patients during their hospital stay and to assess the severity of patient-reported symptoms and functional impairments. Significant differences between patient- and HCP-reported assessment strategies were evaluated.

## Methods

Adult recipients undergoing autologous or allogeneic HSCT were recruited in a single-arm monocentric phase II study assessing the efficacy and safety of a BC–AV-based oral care protocol in addition to standard practice to prevent and treat conditioning regimen-related sOM. Standard practice provides oral and dental hygiene followed by bland saline solution rinses (normal saline or sodium bicarbonate) 3 times per day, increased to 5 per day after OM onset. Two commercially available products, both containing BC and AV, were added to standard practice at each time point. Remargin Colostrum OS® was administered as mouthwash, and Remargin Colostrum Gel® was administered orally to prevent and treat oesophageal mucositis.

The study was designed following the optimal two-stage design by Richard Simon [28]. A first stage of 19 participants was scheduled to evaluate safety, followed by a second stage of 40 patients to complete the planned sample The cutoff for study interruption after the first step was at least 5 participants with sOM.

The World Health Organization (WHO) scale and the Oral Mucositis Daily Questionnaire (OMDQ) [24, 29] were used daily to assess OM from conditioning initiation to day 21 post-transplant or discharge. The OMDQ provided data on mouth and throat soreness (MTS) using a 0–4 Likert scale, where 0 corresponds to “no soreness” and 4 to “extreme soreness”. MTS-activity limitation (MTS-AL) scores regarding swallowing, drinking, eating, talking and sleeping were collected using a 0–4 Likert scale, where 0 is “not limited” and 4 is “unable to do”; the overall-MTS score was assessed on a 0–10 scale, where 0 is “no soreness” and 10 is “worst possible soreness”. The OMDQ made it possible to also collect data on diarrhoea (not reported here). Data on swallowing and talking limitations were also collected by HCPs using the specific parts of the Tardieu scale [30] included in the hospital charts. Clinical and demographic details and data on time of OM onset and duration were collected. Pain was assessed daily by HCPs using 0–10 numerical rating scale.

The Euro Quality of life, 5 dimensions, 3-level tool (EQ-5D-3L) instrument [31] was used to assess QoL at admission (baseline), the day of transplant (T01) and at days + 7 (T07), + 14 (T14), + 21 (T21) and + 28 (T28) post-transplant.

The study protocol was approved by the local ethics committee (n. 2016/0030535, December 28, 2016), and the study was conducted in agreement with the Helsinki Declaration of 1975 and the Guidelines for Good Clinical Practice. All participants gave written informed consent before any study-related procedure took place.

Data are presented as numbers and percentages for categorical variables, and continuous data are expressed as the mean ± standard deviation (SD), unless otherwise specified.

Chi-squared test and Fisher’s exact test were performed to evaluate significant differences in proportions or percentages between the two groups. In particular, Fisher’s exact test was used where the chi-squared test was not appropriate. The test for normal distribution was performed by the Shapiro–Wilk test. The *t* test was used to test differences between two means for paired data. Alternative non-parametric tests, such as Wilcoxon test, were used when the data distribution was not normal. Finally, all tests with *p* value (*p*) < 0.05 were considered significant. Statistical analyses were performed using Matlab statistical toolbox version 2008 (MathWorks, Natick, MA, USA) for 32-bit Windows. The EuroQol Research Foundation user guide was followed to present EQ-5D-3L findings [32]. Three different analyses of the EQ-5D-3L data were done at each time point: the cohort’s health profile description was reported as frequencies and percentages of the 3 levels of perceived problems (no, mild, severe) on mobility, self-care, usual activity, pain/discomfort, anxiety/depression; central tendency measures were used to describe the participants’ overall self-rated health status assessed with the Euro quality of life visual analogue scale (EQ-VAS) on a 0–100 scale; the EQ-5D-3L index was calculated through a time trade-off method and is represented as mean and median values [33].

## Results

Fifty-nine adult HSCT patients (32 [54.2%] males, 27 [45.8%] females) were recruited in the study between the end of 2017 and September 2019; the mean age was 52.4 years (SD ± 12.0; range 18–71). Forty-four of the patients were of working age (74.6%), six were smokers (10.2%), and none of them had alcohol abuse problems. The patients were affected by plasma-cellular disorders (29; 49.2%), acute leukaemia (9; 15.3%), lymphoma (19; 32.2%) or bone marrow failure (2; 3.4%). Forty-four (74.6%) were treated with autologous HSCT and 15 (25.4%) received allograft. Further details on patients’ clinical characteristics are shown in Table 1.

**Table 1:** Patient characteristics

			*n* (%)
Patients	*n*		59
	Age (mean ± SD)		52.4 ± 12.0
	Male		32 (54.2)
	Female		27 (45.8)
Diagnosis	HL/NHL		19 (32.2)
	PD		29 (49.2)
	AL		9 (15.3)
	BMF		2 (3.4)
Transplant	Autologous		44 (74.6)
	Allogeneic		15 (25.4)
		Sibling	8 (13.5)
		Haplo	7 (11.9)
Stem cell source	PBSC		57 (96.6)
	BM		2 (3.4)
Cell product	Cryopreserved		45 (76.3)
	Fresh		14 (23.7)
Conditioning regimen	MAC		56 (94.9)
	RIC		3 (5.1)
Immunosuppression	CsA/MTX		8 (13.5)
	SRL/MPA		7 (11.9)
Growth factors	GCSF	Yes	45 (76.3)
		No	14 (23.7)
	KGF (palifermin)	Yes	0 (0.0)
		No	59 (100.)
Risk factors for OM	Alcohol abuse	Yes	0.0 (0.0)
		No	59 (100)
	Tobacco	Yes	6 (10.2)
		No	53 (89.8)
	Previous OM	Yes	10 (16.9)
		No	49 (83.1)

*n*, number; *SD*, standard deviation; *HL/NHL*, Hodgkin lymphoma/non-Hodgkin lymphoma; *PD*, plasma cell disorders; *AL*, acute leukaemia; *BMF*, bone marrow failure; *PBSC*, peripheral blood stem cells; *BM*, bone marrow; *MAC*, myelo-ablative conditioning; *RIC*, reduced intensity conditioning; *CsA/MTX*, cyclosporine/methotrexate; *SRL/MPA*, sirolimus/mycophenolate acid; *GCSF*, granulocyte-stimulating factor; *KGF*, keratinocyte growth factor; *OM*, oral mucositis

### Healthcare professional-reported outcomes (HCP-ROs)

As reported in our previous work [34], OM was observed in 46 (78.0%) participants; 40 (67.8%) developed mild OM and 6 (10.2%) sOM. Time of OM onset was on an average of 9.1 (SD ± 3.5) days after initiating conditioning regimen, while severe forms occurred on a mean of 11.2 (SD ± 2.9) days after initiation. Duration of OM was on average 10.4 (SD ± 4.3) days, while the duration of sOM was on average 5.2 (SD ± 3.5) days. The average maximum pain score (MPS) was 3.7 (SD ± 2.7).

Data recorded in the hospital charts showed that the ability to swallow was altered in 39 (66.1%) participants starting on an average of 11.4 (SD ± 3.8) days after starting chemotherapy; the mean duration of this problem was 6.2 (SD ± 3.2) days. Difficulty talking was recorded in the hospital chart of 27 (45.8%) participants; the mean time of onset was 12.1 (SD ± 3.8) days later, and duration was an average of 5.0 (SD ± 3.4) days.

### Patient-reported outcome measures (PROMs)

#### OMDQ

Fifty-one recipients (86.4%) reported mouth and throat soreness (MTS): 22 (37.3%) patients reported mild MTS (score 1–2), while 29 (49.1%) reported severe MTS (score 3–4). Oral symptoms occurred on average 7.9 (SD ± 4.0) days after the initiation of conditioning, while severe MTS began on average 11.9 (SD ± 3.8) days after. Symptom duration was an average of 10.5 (SD ± 4.6) days, while severe MTS duration was an average of 4.4 (SD ± 2.5) days. Swallowing was altered in 50 (84.7%) patients; drinking limitations were present in 45 (76.3%) patients, and 46 (78.0%) reported eating problems. Oral condition-related difficulty talking and sleep disturbances were reported by 32 (54.2%) and 21 (35.6%) participants, respectively. Maximum overall-MTS was on average 6.1 (SD ± 2.3).

Participants reported that swallowing issues began on average 8.8 (SD ± 4.3) days from the start of conditioning, and its mean duration was 9.3 (SD ± 4.4) days. Difficulty talking started an average of 9.0 (SD ± 4.0) days after the initiation of conditioning and lasted an average of 7.4 (SD ± 6.3) days.

#### Comparisons

Comparing the patient-reported outcomes with those of the HCPs (Table 2), no significant difference was found in the number of patients developing oral problems (*p* = 0.23); OMDQ allowed detection of oral symptom onset sooner compared to the WHO scale (*p* = 0.025), although no difference was found for the duration of oral problems. The overall highest patient-related MTS score was significantly higher than that of the HCPs using the NRS scale (*p* < 0.0001). A significant difference was found between the MTS and the WHO severity grading (*p* < 0.0001).

**Table 2:** Comparison of PROMs and HCP-ROs

**Parameters**	**PROMs**	**HCP-ROs**	***p* value (test)**
Patients with oral problems	MTS	WHO	
*% (n)*	86.4 (51)	78.0 (46)	0.23 (C)
No problem (grade 0)	13.6 (8)	22.0 (13)	
Mild (grades 1–2)	37.3 (22)	67.8 (40)	< 0.0001 (C)*
Severe (grades 3–4)	49.1 (29)	10.2 (6)	
Onset time (days)			
Mean ± SD	7.9 ± 4.0	9.1 ± 3.5	0.025 (T)*
Median (IQR)	8.0 (5.0–10.0)	9.0 (7.0–11.0)	
Data distribution	aN	aN	
Duration (days)			
Mean ± SD	10.5 ± 4.6	10.4 ± 4.3	0.20 (T)
Median (IQR)	11.0 (7.5–13.0)	10.0 (7.0–13.0)	
Data distribution	aN	aN	
Patients with symptoms	Overall-MTS	NRS	
*% (n)*	86.4 (51)	86.4 (51)	1 (C)
Symptoms highest score			
Mean ± SD	5.3 ± 3.0	3.7 ± 2.7	
Median (IQR)	6.0 (3.0–8.0)	4.0 (2.0–5.0)	< 0.0001 (W)*
Data distribution	rN	rN	

*SD*, standard deviation; *IQR*, interquartile range; *T*, t test; *aN*, accept normality; *rN*, reject normality; *C*, chi-square test; *W*, Wilcoxon test

^*^Significant test

#### Quality of life

Descriptive health profiles of the sample are shown in Table 3, where the 3 levels of perceived alterations (no problem, mild problems, severe problems) at each time point are reported. Excluding mobility, percentages of the activities that were worse on day + 7 post-transplant compared to baseline values were observed, in particular regarding pain (69.5% vs 39.0% of patients referring pain).

**Table 3:** EQ-5D-3L: cohort’s health profile at each time point

EQ-5D-3L levels	EQ-5D-3L dimensions				
**Time point (*n*)**	**Mobility % (*n*)**	**Self-care % (*n*)**	**Usual activities % (*n*)**	**Pain/discomfort % (*n*)**	**Anxiety/depression % (*n*)**
Baseline (*n* = 59)					
Level 1	89.8 (53)	96.6 (57)	83.1 (49)	61.0 (36)	78.0 (46)
Level 2	10.2 (6)	3.4 (2)	16.9 (10)	39.0 (23)	22.0 (13)
Level 3	0.0 (0)	0.0 (0)	0.0 (0)	0.0 (0)	0.0 (0)
T01—HSCT (*n* = 59)					
Level 1	89.8 (53)	91.5 (54)	88.1 (52)	64.4 (38)	81.4 (48)
Level 2	8.5 (5)	6.8 (4)	8.5 (5)	35.6 (21)	16.9 (10)
Level 3	1.7 (1)	1.7 (1)	3.4 (2)	0.0 (0)	1.7 (1)
T07 (*n* = 59)					
Level 1	89.8 (53)	88.1 (52)	81.4 (48)	30.5 (18)	72.9 (43)
Level 2	6.8 (4)	5.1 (3)	8.5 (5)	62.7 (37)	23.7 (14)
Level 3	3.4 (2)	6.8 (4)	10.1 (6)	6.8 (4)	3.4 (2)
T14 (*n* = 59)					
Level 1	89.8 (53)	78.0 (46)	71.2 (42)	52.5 (31)	76.3 (45)
Level 2	3.4 (2)	15.2 (9)	22.0 (13)	40.7 (24)	22.0 (13)
Level 3	6.8 (4)	6.8 (4)	6.8 (4)	6.8 (4)	1.7 (1)
T21 (*n* = 27)					
Level 1	70.4 (19)	66.7 (18)	63.0 (17)	63.0 (17)	88.9 (24)
Level 2	29.6 (8)	18.5 (5)	22.2 (6)	29.6 (8)	11.1 (3)
Level 3	0.0 (0)	14.8 (4)	14.8 (4)	7.4 (2)	0.0 (0)
T28 (*n* = 6)					
Level 1	83.3 (5)	66.7 (4)	50.0 (3)	33.3 (2)	66.7 (4)
Level 2	16.7 (1)	33.3 (2)	33.3 (2)	66.7 (4)	33.3 (2)
Level 3	0.0 (0)	0.0 (0)	16.7 (1)	0.0 (0)	0.0 (0)

**Fig. 1:**
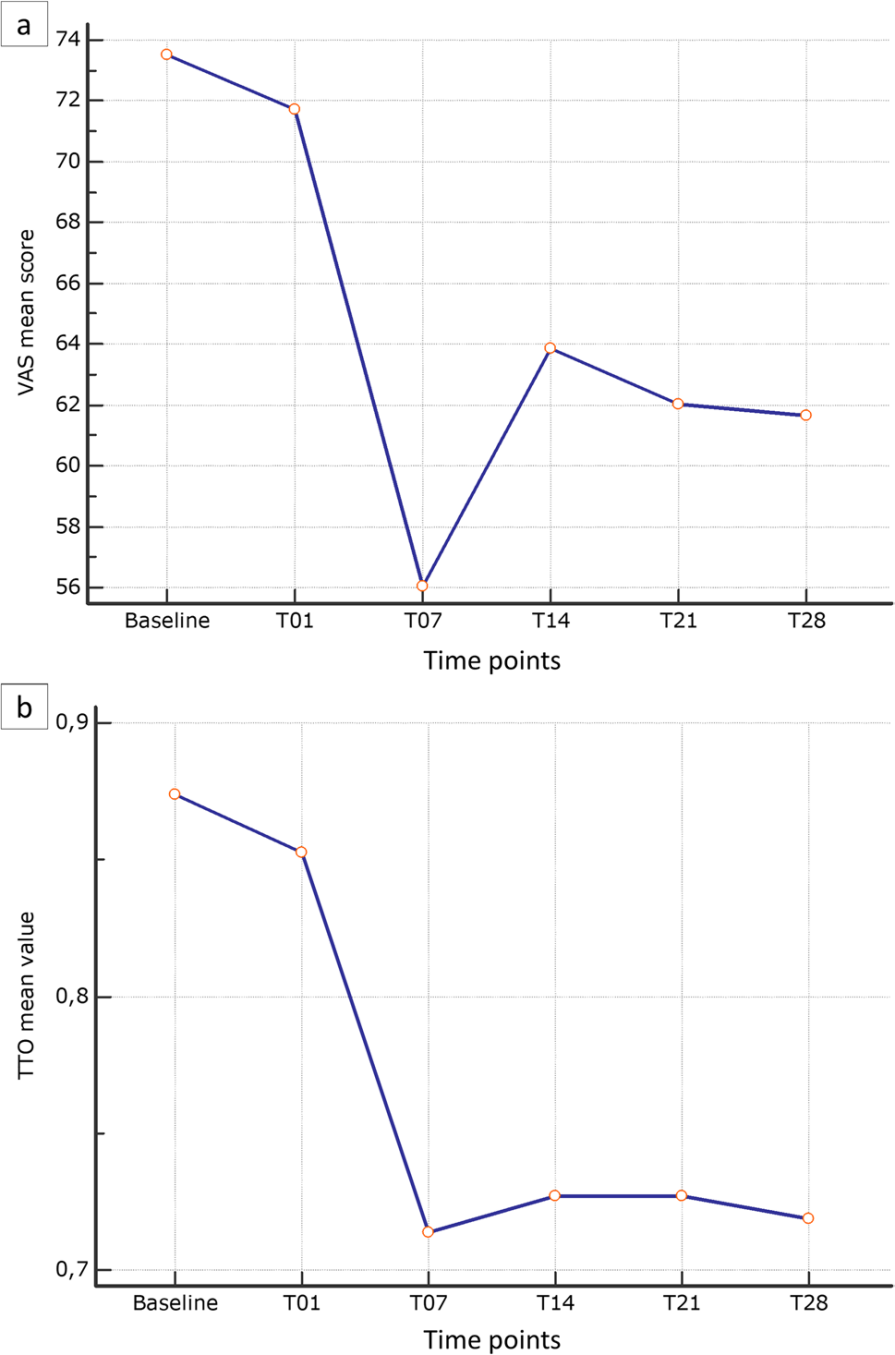
EQ-VAS mean score and EQ-5D-3L index mean score variations

The analysis of the overall self-rated health status assessed using the EQ-VAS score showed that general health status worsened, with a significant difference between baseline mean value and day + 14 post-transplant (*p* < 0.0001), with the nadir on day + 7 (mean 56.1; SD ± 18.8) followed by a slight improvement on day + 14. As shown in Table 4, many comparisons of the mean rank value at various time points proved to be significant, in particular those including day + 7 values. The same trend was observed for the EQ-5D-3L index; a significant difference between baseline and day + 14 (*p* = 0.0006) was found, as was the worsening of patient-reported general condition between baseline and day + 7 post-transplant (mean 0.714 ± 0.288). Figure 1 a and b) report both EQ-VAS mean score and EQ-5D-3L index mean score variations. The inferential analysis did not include T21 and T28 due to patient discharge from hospital.

**Table 4:** EQ-5D-3L visual analogue scale and index

EQ-5D-3L	VAS			Index		
	*n* (aN-rN)	Mean ± SD	Median (IQR) mean rank	*n* (aN-rN)	Mean ± SD	Median (IQR) mean rank
Baseline	59 (rN)	73.5 ± 18.8	80 (60–90) 2.92	59 (rN)	0.834 ± 0.132	0.848 (0.796–1.0) 2.83
T01	59 (rN)	71.7 ± 20.3	80 (52.5–90) 2.85	59 (rN)	0.853 ± 0.191	0.848 (0.796–1.0) 2.73
T07	59 (aN)	56.1 ± 21.4	60 (50–70) 1.75	59 (rN)	0.714 ± 0.288	0.796 (0.725–0.840) 2.14
T14	59 (rN)	63.9 ± 22.5	60 (50–80) 2.48	59 (rN)	0.727 ± 0.348	0.796 (0.689–1.0) 2.31
T21	27 (aN)	62.0 ± 18.8	60 (50–80) —	27 (rN)	0.727 ± 0.346	0.796 (0.613–1.0) —
T28	6 (aN)	61.7 ± 19.7	60 (55–80) —	6 (aN)	0.719 ± 0.299	0.743 (0.639–1.0) —
*p* value^¥^	Baseline-T14 *p* < 0.0001 (AF)*			Baseline-T14 *p* = 0.0006 (AF)*		
Mean rank comparison^¥^	T07 < baseline, *p* = 0.05 (Co)*			T07 < baseline, *p* = 0.05 (Co)*		
	T07 < T01, *p* = 0.0041 (Co)*			T07 < T01, *p* = 0.0041 (Co)*		
	T07 < T14, *p* = 0.0001 (Co)*			T14 < baseline, *p* = 0.0001 (Co)*		
	Baseline > T14, *p* < 0.05 (Co)*			T14 < T01, *p* < 0.05 (Co)*		

*SD*, standard deviation; *IQ*, interquartile range; *aN*, accept normality; *rN*, reject normality; *AF*, ANOVA Friedman test; *Co*, Conover post hoc Friedman test

^*^Significant test

^¥^T21 and T28 excluded by the analysis due to patients lost (hospital discharging)

## Discussion

This paper reports the secondary findings of a phase II study on the efficacy of an experimental BC–AV-based oral care protocol to prevent and treat OM in patients undergoing HSCT (autologous or allogeneic grafts). A first work compared this experimental approach with a historical control group [27]. The study collected OM-related PROMs in addition to HCP-ROs. In this second study, PROMs were described and compared with those collected and managed by HCPs. In addition, the QoL findings presented here highlight the results regarding the participants’ health profile description, their perceived general health status and EQ-5D-3L index variations among repeated measures.

PROMs allow patients to evaluate their health without any external HCP influence [34]. When used in clinical practice, these tools can be coupled with other clinical assessment instruments, which together provide a more accurate representation of reality [35]. The importance of patients’ perception of measures to understanding the impact of health on daily living and to modulating healthcare actions has been well described [36, 37], and PROMs have been recommended in clinical trials evaluating new strategies to OM prevention and treatment [38]. Diagnosis, treatment and various aspects of the patient-HCP relationship, such as communication issues, patient compliance and clinical outcomes, benefit from the use of PROMs [39, 40]. OM and its related pain, including physical, emotional, functional and psychological impairments, may have a dramatic impact on various aspects of HSCT patients’ QoL [41].

As previously demonstrated by Stiff and colleagues [24], our findings show that the use of OMDQ made it possible to detect oral problems at least 1 day sooner compared to the clinical assessment, while the duration of oral symptoms was similar. As reported by other authors [21], the implementation of OMDQ in clinical practice may foster a proactive approach by HCPs managing OM. In our study, OMDQ allowed us to identify some patients with mild MTS who were undetected by the WHO scale. Significant differences between the two assessment methods were found on OM severity grading distribution and on OM-related pain scores. These differences could be associated with various patient-related factors, including anxiety, depression, individual pain tolerance and difficulty coping with the emerging changes. They could also be due to HCP-related issues, such as the tendency to underestimate referred symptoms or difficulty maintaining competence in the use of the assessment scales. All HCPs who assessed OM and its related symptoms in this study regularly received training on the use of the WHO scale and other assessment tools. In addition, they assessed OM as they routinely do in their clinical practice and were not informed of the study objectives. However, a study effect cannot be excluded. For these reasons, a double-blind placebo-controlled randomised trial is being set up at our research hospital.

The trend of OM severity during HSCT has been well described in the literature. In the majority of affected patients, it increases rapidly after the initiation of conditioning, reaching the highest score on day + 7 post-transplant, then tends to decrease more slowly until day + 28 [42]. For this reason, and considering the pathobiological definition of OM [43], the collected OM data refer to the first day of conditioning and not to the day of transplantation, as in most studies. An interesting finding of our study was the description of both EQ-VAS and EQ-5D-3L Index score variations during the HSCT pathway, which highlighted a clear correlation with the trend of OM severity reported by Spielberg and colleagues [44] (Fig. 1 a and b), with the nadir of QoL scores at day + 7 post-transplant. Although OM is considered one of the worst toxic effects of the conditioning regimens [20], few studies have provided any information about the link between QoL and OM in HSCT patients [44], and only one study has attempted to understand the patients’ experience when affected by OM [45]. Further quantitative and qualitative studies are necessary to explore patients’ perception and to enhance its role in daily practice.

## Conclusions

This work provides some findings on the relationship between PROMs and HCP-ROs in the setting of a phase II study exploring the efficacy and safety of a BC–AV-based oral care protocol to prevent and treat OM in HSCT patients. Compared to the HCPs’ assessments using the WHO and NRS scales, the OMDQ detected OM earlier, and its severity and related pain were significantly greater. The variations in the QoL measured using EQ-5D-3L instrument (EQ-VAS and EQ-5D-3L Index) were clearly correlated with the severity of OM during the hospital stay. A randomised controlled trial is being set up at our institution to confirm the efficacy of the experimental oral care protocol and to shed light on what has been reported here.

## Data Availability

The datasets generated during the current study are available from the corresponding author on reasonable request.

## References

[1] Granot N, Storb R (2020). History of hematopoietic cell transplantation: challenges and progress. Haematologica.

[2] Zulu S, Kenyon M, Principles of conditioning therapy and cell infusion, Kenyon M, Babic A (2017). 22. The European blood and marrow transplantation textbook for nurses: under the auspices of EBMT.

[3] Copelan EA (2006). Hematopoietic stem-cell transplantation. N Engl J Med.

[4] Vagliano L, Feraut C, Gobetto G, Trunfio A, Errico A, Campani V (2011). Incidence and severity of oral mucositis in patients undergoing haematopoietic SCT—results of a multicentre study. Bone Marrow Transplant.

[5] Filicko J, Lazarus HM, Flomenberg N (2003). Mucosal injury in patients undergoing hematopoietic progenitor cell transplantation: new approaches to prophylaxis and treatment. Bone Marrow Transplant.

[6] Chaudhry HM, Bruce AJ, Wolf RC (2016). The incidence and severity of oral mucositis among allogeneic hematopoietic stem cell transplantation patients: a systematic review. Biol Blood Marrow Transplant.

[7] Bachour PC, Sonis ST (2018). Predicting mucositis risk associated with cytotoxic cancer treatment regimens: rationale, complexity, and challenges. Curr Opin Support Palliat Care.

[8] Sonis ST (2009). Mucositis: the impact, biology and therapeutic opportunities of oral mucositis. Oral Oncol.

[9] Borbasi S, Cameron K, Quested B, Olver I, To B, Evans D. 2002 More than a sore mouth: patients’ experience of oral mucositis. In: *Oncol Nurs Forum*. Vol 29 10.1188/02.ONF.1051-1057 12183754

[10] Rodrigues-Oliveira L, Kowalski LP, Santos M (2021). Direct costs associated with the management of mucositis: a systematic review. Oral Oncol.

[11] Bezinelli LM, Eduardo FP, Neves VD, Correa L, Lopes RM, Michel-Crosato E, Hamerschlak N, Biazevic MG (2016). Quality of life related to oral mucositis of patients undergoing haematopoietic stem cell transplantation and receiving specialised oral care with low-level laser therapy: a prospective observational study. Eur J Cancer Care (Engl).

[12] Miranda-Silva W, Gomes-Silva W, Zadik Y (2021). MASCC/ISOO clinical practice guidelines for the management of mucositis: sub-analysis of current interventions for the management of oral mucositis in pediatric cancer patients. Support Care Cancer.

[13] Mawardi H, Treister N, Felemban O (2021). Current practice of oral care for hematopoietic stem cell transplant patients: a survey of the Eastern Mediterranean Blood and Marrow transplantation group.

[14] Rathe M, De Pietri S, Wehner PS (2020). Bovine colostrum against chemotherapy-induced gastrointestinal toxicity in children with acute lymphoblastic leukaemia: a randomized, double-blind, placebo-controlled trial. J Parenter Enter Nutr.

[15] da LimaSilva ICG, deFátimaSoutoMaior L, Gueiros LAM, Leão JC, Higino JS, Carvalho AAT (2021). Clinical applicability of natural products for prevention and treatment of oral mucositis: a systematic review and meta-analysis. Clin Oral Investig.

[16] Yarom N, Hovan A, Bossi P, et al. 2020 Systematic review of natural and miscellaneous agents, for the management of oral mucositis in cancer patients and clinical practice guidelines—part 2: honey, herbal compounds, saliva stimulants, probiotics, and miscellaneous agents. *Support Care Cancer* Published online 1–16. 10.1007/s00520-019-05256-4 32056010

[17] Gok Metin Z, Helvaci A, Gulbahar EM (2021). Effects of aloe vera in adults with mucocutaneous problems: a systematic review and meta-analysis. J Adv Nurs.

[18] Guberti M, Botti S, Capuzzo MT (2021). Bovine colostrum applications in sick and healthy people: a systematic review. Nutrients.

[19] Salehi B, Lopez-Jornet P, Pons-Fuster López E (2019). Plant-derived bioactives in oral mucosal lesions: a key emphasis to curcumin, lycopene, chamomile, aloe vera, green tea and coffee properties. Biomolecules.

[20] Bellm LA, Epstein JB, Rose-Ped A, Martin P, Fuchs HJ (2000). Patient reports of complications of bone marrow transplantation. Support Care Cancer.

[21] Quinn B, Potting CM, Stone R, Blijlevens NM, Fliedner M, Margulies A, Sharp L (2008). Guidelines for the assessment of oral mucositis in adult chemotherapy, radiotherapy and haematopoietic stem cell transplant patients. Eur J Cancer.

[22] Bateman E, Keefe D (2011). Patient-reported outcomes in supportive care. Semin Oncol.

[23] Stiff PJ, Emmanouilides C, Bensinger WI (2006). Palifermin reduces patient-reported mouth and throat soreness and improves patient functioning in the hematopoietic stem-cell transplantation setting. J Clin Oncol.

[24] Stiff PJ, Erder H, Bensinger W (2006). Reliability and validity of a patient self-administered daily questionnaire to assess impact of oral mucositis (OM) on pain and daily functioning in patients undergoing autologous hematopoietic stem cell transplantation (HSCT). Bone Marrow Transplant.

[25] Cheng KK, Lee V, Li CH, Yuen HL, Epstein JB (2012). Oral mucositis in pediatric and adolescent patients undergoing chemotherapy: the impact of symptoms on quality of life. Support Care Cancer.

[26] Kashiwazaki H, Matsushita T, Sugita J, Shigematsu A, Kasashi K, Yamazaki Y, Kanehira T, Yamamoto S, Kondo T, Endo T, Tanaka J, Hashino S, Nishio M, Imamura M, Kitagawa Y, Inoue N (2012). Professional oral health care reduces oral mucositis and febrile neutropenia in patients treated with allogeneic bone marrow transplantation. Support Care Cancer.

[27] The 47th Annual Meeting of the European Society for Blood and Marrow Transplantation: Nurses Group – Oral Session (NO001 – NO010). Bone Marrow Transplant 56, 336–343 (2021). doi: 10.1038/s41409-021-01344-4.

[28] Simon R (1989). Optimal two-stage designs for phase II clinical trials. Control Clin Trials.

[29] Syrjala K, Hays RD, Kallich JD (2003). Impact of oral mucositis and its sequelae on quality of life. Blood.

[30] Tardieu C, Cowen D, Thirion X, Franquin JC (1996). Quantitative scale of oral mucositis associated with autologous bone marrow transplantation. Eur J Cancer B Oral Oncol.

[31] Rabin R, de Charro F (2001). EQ-5D: a measure of health status from the EuroQol Group. Ann Med.

[32] EuroQol Research Foundation. EQ-5D-3L User Guide, 2018. Available from: https://euroqol.org/publications/user-guides. Accessed: January 10, 2022.

[33] Scalone L, Cortesi PA, Ciampichini R, Belisari A, D’Angiolella LS, Cesana G, Mantovani LG. Italian population-based values of EQ-5D health states. Value Health. 2013 Jul-Aug;16(5):814–22. doi: 10.1016/j.jval.2013.04.008. 10.1016/j.jval.2013.04.008 23947975

[34] Basch E, Torda P, Adams K (2013). Standards for patient-reported outcome-based performance measures. JAMA.

[35] Ní Ríordáin R, Shirlaw P, Alajbeg I, Al Zamel GY, Fung PL, Yuan AD, McCreary C, Stoopler ET, De Rossi SS, Lodi G, Greenberg MS, Brennan MT (2015). World Workshop on Oral Medicine VI: patient-reported outcome measures and oral mucosal disease: current status and future direction. Oral Surg Oral Med Oral Pathol Oral Radiol.

[36] Bowling A (1995). Measuring disease: a review of disease specific quality of life measurement scales.

[37] Black N, Jenkinson C (2009). Measuring patients’ experiences and outcomes. BMJ.

[38] Bellm LA, Cunningham G, Durnell L, Eilers J, Epstein JB, Fleming T, Fuchs HJ, Haskins MN, Horowitz MM, Martin PJ, McGuire DB, Mullane K, Oster G (2002). Defining clinically meaningful outcomes in the evaluation of new treatments for oral mucositis: oral mucositis patient provider advisory board. Cancer Invest.

[39] Valderas JM, Kotzeva A, Espallargues M, Guyatt G, Ferrans CE, Halyard MY, Revicki DA, Symonds T, Parada A, Alonso J (2008). The impact of measuring patient-reported outcomes in clinical practice: a systematic review of the literature. Qual Life Res.

[40] Marshall S, Haywood K, Fitzpatrick R (2006). Impact of patient-reported outcome measures on routine practice: a structured review. J Eval Clin Pract.

[41] Alvarado Y, Bellm LA, Giles FJ (2002). Oral mucositis: time for more studies. Hematology.

[42] Spielberger R, Stiff P, Bensinger W, Gentile T, Weisdorf D, Kewalramani T, Shea T, Yanovich S, Hansen K, Noga S, McCarty J, LeMaistre CF, Sung EC, Blazar BR, Elhardt D, Chen MG, Emmanouilides C (2004). Palifermin for oral mucositis after intensive therapy for hematologic cancers. N Engl J Med.

[43] Sonis ST (2004). The pathobiology of mucositis. Nat Rev Cancer.

[44] Al-Rudayni A, Gopinath D, Maharajan M, Menon R (2020). Impact of oral mucositis on quality of life in patients undergoing oncological treatment: a systematic review. Transl Cancer Res.

[45] Kamulegeya A, Nakanjako D, Orem J, Mayanja-Kizza H (2021). Experiences of patients who developed oral mucositis during solid neoplasms treatment: a Ugandan qualitative study. J Patient Rep Outcomes.

